# Supplementary material for: Dietary Intakes and Eating Behavior between Metabolically Healthy and Unhealthy Obesity Phenotypes in Asian Children and Adolescents
Source: Nutrients. 2022 Nov 12;14(22):4796. doi: 10.3390/nu14224796 (PMC9697734; doi:10.3390/nu14224796)
Supplement: Supplementary file 1 [file nutrients-14-04796-s001.zip › nutrients-1967112-supplementary.pdf]

Table S1. Acceptable macronutrient distribution range (AMDR) and recommended dietary allowance (RDA) for individuals of different age groups

		Female				Male			
	**Source**	**4-8 years old**	**9-13 years old**	**14-18 years old**	**19-30 years old**	**4-8 years old**	**9-13 years old**	**14-18 years old**	**19-30 years old**
**Dietary Guidelines for Americans 2020–2025**									
Carbohydrates (% kcal)	AMDR	45-65	45-65	45-65	45-65	45-65	45-65	45-65	45-65
Total fat (% kcal)	AMDR	25-35	25-35	25-35	20-35	25-35	25-35	25-35	20-35
Saturated fat (% kcal)	AMDR	<10	<10	<10	<10	<10	<10	<10	<10
Protein (% kcal)	AMDR	10-30	10-30	10-30	10-35	10-30	10-30	10-30	10-35
Dietary fiber (g/1000 kcal)	RDA	17	22	25	28	20	25	31	34

		Female					Male				
	**Source**	**7-9 years old**	**10-11 years old**	**12-15 years old**	**16-17 years old**	**18-29 years old**	**7-9 years old**	**10-11 years old**	**12-15 years old**	**16-17 years old**	**18-29 years old**
**Health Promotion Board, Singapore**											
Calcium (mg)^	RDA	700	1000	1000	1000	1000*/800	700	1000	1000	1000	1000*/800
Iron (mg)^	RDA	7	7	18	19	18	7	7	12	6	8
Vitamin A (mcg)^	RDA	400	575	725	750	750	400	575	725	750	750

Table S2. Food items in the different food groups

Food groups	Food items
Deep fried food	Dimsum fried (wantan, carrot cake, mantou, spring roll, you tiao, breaded chicken bun, etc), chicken and pork deep fried (KFC fried chicken, battered/ breaded/ flour and fried), fishcake fried, fishball fried, tofu fried, potato cutlet, egg fried, seafood deep fried (prawns, soft shell crab, etc), oily and non-oily fish (battered fish)
Fast food and processed convenience food	Fried potatoes (french fries, hash brown, etc), pizza, burger, nuggets, popcorn chicken, patty, food from convenience store, frozen-ready meal
Fish	Fish cooked by dry heat or steamed, canned tuna, sardines, salmon (Sashimi, grilled)
Fruits	Dried fruits (raisins), guava, dragonfruit, melon, tropical fruit (pineapple, mangoes, etc), stone fruit, grapes, berries, orange, papaya, banana, apples, pears
Savory snacks	Corn chips (with cheese, etc), potato chips, seaweed, pies and puffs (curry puff, chicken pie, sausage rolls, etc), popiah, crackers, papadum
Sugar-sweetened beverage (SSB)	Milo, horlicks, chocolate drinks, cultured drinks (cultured milk, yogurt drinks, etc), carbonated drinks, sweetened drinks, low calorie drinks, sports drinks, soya milk, traditional drinks (chrysanthemum tea), coffee and tea (3-in-1 powder, coffee/ tea with condensed milk or sugar, bubble tea with/ without toppings, etc), cordial (rose syrup, blueberry, lime, etc), fruit juice, drinks with natural sugar, flavoured milk
Sweet snacks	Ice cream, jellies, puddings, sweets, chocolates, fried doughy snacks, biscuits, cookies, puff pastries, cakes (cheesecakes, idli, chiffon cake, etc), dessert (green bean soup, etc)
Vegetables	Mushroom, gourd, lentils, stalk vegetables, broccoli, cauliflower, pale and dark leafy vegetables (lettuce, spinach, etc), tomato, red and green peppers, corn, peas, beans, carrots, onions, radish, pumpkin, coleslaw, mashed potato, lotus roots, yam, bamboo shoots
Whole grains	Unpolished rice (brown rice), unpolished rice porridge, oats, wholemeal bread

Table S3. Food groups, nutrients intakes, and eating behavior between children/adolescents with MHO and children/adolescents with MUO by MS definition

	MS definition		
	**MHO (n=42)**	**MUO (n=10)**	***p***
**Food groups (continuous variables)**			
Deep fried food (g)	60.5 (23.7-127)	52.3 (42.0-150)	0.990
Fast food and processed convenience food (g)	75.3 (0.00-147)	73.7 (0.00-142)	0.692
Fish (g)	0.00 (0.00-20.5)	35.1 (0.00-80.0)	0.228
Fruits (g)	0.00 (0.00-44.8)	0.00 (0.00-35.7)	0.835
Savory snacks (g)	6.22 (0.00-28.6)	0.00 (0.00-72.8)	0.878
Sugar-sweetened beverage, SSB (ml)	307 (173-436)	278 (148-558)	0.785
Sweet snacks (g)	25.1 (0.00-74.2)	24.9 (8.00-56.6)	0.674
Vegetables (g)	77.4 (41.9-124)	89.7 (32.0-169)	0.477
Whole grains (g)	0.00 (0.00-18.3)	4.83 (0.00-79.2)	1.000
**Nutrients (continuous variables)**			
Total energy (kcal)	1880 (1680-2360)	1820 (1730-2220)	0.553
Carbohydrates (% kcal)	47.6 (42.0-52.3)	47.5 (39.3-51.7)	0.471
Protein (% kcal)	17.2 (15.0-20.4)	17.4 (14.9-21.9)	0.495
Total fat (% kcal)	35.0 (32.2-39.1)	36.0 (31.6-40.0)	0.632
Saturated fat (% kcal)	12.2 (11.0-14.3)	13.0 (10.4-14.7)	0.252
Monounsaturated fat (% kcal)	12.0 (10.2-14.8)	13.6 (12.4-14.8)	0.570
Polyunsaturated fat (% kcal)	6.54 (5.20-8.11)	6.35 (5.63-7.21)	0.606
Beta-carotene (mcg per 1000 kcal)	0.86 (0.00-13.3)	0.10 (0.00-0.94)	0.784
Calcium (mg per 1000 kcal)	276 (209-351)	219 (204-284)	0.565
Cholesterol (mg per 1000 kcal)	190 (147-226)	201 (129-281)	0.778
Dietary fibre (g per 1000kcal)	7.03 (5.99-7.93)	6.19 (5.59-6.68)	0.682
Iron (mg per 1000 kcal)	6.36 (5.23-7.25)	6.01 (4.95-6.70)	0.552
Sodium (mg per 1000 kcal)	1640 (1380-2040)	1700 (1400-1930)	0.823
Vitamin A (mcg per 1000 kcal)	275 (155-363)	207 (170-349)	0.410
**% of participants meeting AMDR/RDA of nutrients**			
Carbohydrates† (AMDR)	59.5	60	0.740
Total fat† (AMDR)	50	30	0.369
Saturated fat† (AMDR)	16.7	20	0.956
Protein† (AMDR)	100	100	1.000
Calcium‡ (RDA)	7.1	0	0.998
Dietary fiber† (RDA)	7.1	0	0.999
Iron‡ (RDA)	61.9	40	0.136
Vitamin A‡ (RDA)	31	10	0.276
**Eating behavior (continuous variables)**			
Cognitive dietary restraint	15.5 (13.8-18.0)	14 .0 (12.8-17.0)	0.428
Emotional eating	6.00 (4.00-8.00)	5.50 (3.75-6.25)	1.000
Uncontrolled eating	21.0 (18.8-24.3)	20.5 (18.0-22.3)	0.130

Data were presented as median (interquartile range: 25th-75th percentile) and percentage (%) for continuous and categorical variables respectively. Differences in continuous variables between groups were analyzed using quantile regression with adjustment for age, sex, race and BMI-SDS, while differences in categorical variables between groups were analyzed using logistic regression with adjustment for age, sex, race and BMI-SDS. † AMDR and RDA of nutrients were according to Dietary Guidelines for Americans 2020-2025, ‡RDA of nutrients were according to dietary guidelines by Health Promotion Board, Singapore.

Table S4. Food groups, nutrient intakes, and eating behavior between children/adolescents with MHO and children/adolescents with MUO (MS definition) stratified by sex

	MS definition					
	Male			Female		
	MHO (n=25)	MUO (n=6)	*p*	MHO (n=17)	MUO (n=4)	*p*
**Food groups**						
Deep fried food (g)	62.2 (39.8-136)	88.1 (44.6-155)	0.436	46.7 (21.2-117)	47.9 (37.5-169)	0.675
Fast food and processed convenience food (g)	90.0 (9.33-151)	73.7 (0.00-132)	0.539	50.0 (0.00-116)	70.5 (0.00-144)	0.902
Fish (g)	0.00 (0.00-64.2)	36.4 (0.00-80.0)	0.105	0.00 (0.00-8.10)	35.1 (5.61-117)	0.493
Fruits (g)	0.00 (0.00-38.4)	0.00 (0.00-10.5)	0.782	4.00 (0.00-60.0)	16.8 (0.00-163)	0.707
Savory snacks (g)	0.00 (0.00-25.0)	0.00 (0.00-46.6)	0.957	12.8 (0.00-31.8)	45.5 (0.00-125)	0.317
Sugar-sweetened beverage, SSB (ml)	317 (162-472)	253 (118-630)	0.256	300 (145-416)	487 (223-595)	0.219
Sweet snacks (g)	25.2 (0.00-79.3)	15.3 (0.00-33.5)	0.610	23.3 (0.00-91.7)	50.8 (30.1-69.2)	0.525
Vegetables (g)	69.3 (30.1-109)	89.7 (47.1-169)	0.534	85.3 (50.2-151)	60.8 (6.17-310)	0.538
Whole grains (g)	0.00 (0.00-18.5)	42.3 (7.25-117)	0.117	0.00 (0.00-10.0)	0.00 (0.00-0.00)	1.000
**Nutrients**						
Total energy (kcal)	2240 (1750-2420)	1820 (1660-2220)	0.089	1780 (1630-2020)	1800 (1730-3130)	0.872
Carbohydrates (% kcal)	49.7 (43.3-52.5)	48.8 (36.3-55.5)	0.829	44.0 (40.3-50.8)	45.1 (40.8-49.6)	0.990
Protein (% kcal)	16.6 (15.0-18.8)	17.4 (15.5-22.6)	0.825	19.7 (14.9-21.3)	17.1 (13.4-21.6)	0.232
Total fat (% kcal)	34.5 (32.1-38.5)	34.2 (28.5-41.1)	0.573	36.2 (32.2-40.7	37.2 (36.0-39.4)	0.651
Saturated fat (% kcal)	11.7 (9.86-13.4)	11.9 (9.94-15.2)	0.454	12.4 (12.0-15.7)	13.2 (12.4-14.6)	0.901
Monounsaturated fat (% kcal)	12.0 (10.3-14.8)	12.9 (12.0-14.5)	0.993	13.2 (9.61-14.7)	14.4 (13.0-15.3)	0.292
Polyunsaturated fat (% kcal)	6.58 (5.18-8.31)	6.05 (4.87-7.26)	0.215	6.46 (5.37-7.21)	6.86 (6.02-7.42)	0.616
Beta-carotene (mcg per 1000 kcal)	0.00 (0.00-3.89)	0.48 (0.00-9.78)	0.982	5.16 (0.18-33.9)	0.00 (0.00-0.61)	0.942
Calcium (mg per 1000 kcal)	248 (181-304)	213 (193-284)	0.966	299 (252-383)	228 (197-284)	0.604
Cholesterol (mg per 1000 kcal)	181 (149-226)	236 (127-281)	0.105	200 (142-226)	184 (123-342)	0.591
Dietary fiber (g per 1000kcal)	7.00 (5.90-8.27)	6.28 (5.59-6.99)	0.215	7.11 (6.02-7.57)	6.19 (5.45-6.61)	0.729
Iron (mg per 1000 kcal)	6.23 (5.11-7.26)	5.64 (4.95-6.94)	0.494	6.55 (5.30-7.25)	6.27 (4.14-6.62)	0.400
Sodium (mg per 1000 kcal)	1580 (1420-2050)	1700 (1410-1880)	0.800	1840 (1340-2160)	1660 (1330-2020)	0.940
Vitamin A (mcg per 1000 kcal)	219 (112-329)	207 (119-349)	0.932	323 (234-403)	264 (187-385)	0.193
**Eating behavior**						
Cognitive dietary restraint	15.0 (13.0-17.0)	13.5 (12.0-14.8)	0.144	17.0 (14.0-18.5)	16.5 (13.8-18.5)	0.238
Emotional eating	6.00 (4.00-7.00)	5.00 (3.75-6.00)	0.450	6.00 (4.00-8.00)	6.50 (3.75-7.75)	0.830
Uncontrolled eating	22.0 (18.0-25.0)	21.0 (19.8-22.5)	0.467	21.0 (19.5-23.5)	18.0 (17.3-21.8)	0.260

Data were presented as median (interquartile range: 25th-75th percentile). Differences in continuous variables between groups were analyzed using quantile regression with adjustment for age, race and BMI-SDS.

Table S5. Food groups, nutrient intakes, and eating behavior between the 3 ethnic groups

	Chinese (n=21)	Malay (n=28)	Indian (n=3)	*p*
**Food groups**				
Deep fried food (g)	62.2 (42.0-137)	52.0 (23.5-126)	94.4	0.500
Fast food and processed convenience food (g)	50.0 (0.00-125)	81.5 (16.1-170)	40.0	0.301
Fish (g)	0.00 (0.00-62.6)	0.00 (0.00-53.0)	0.00	0.874
Fruits (g)	14.1 (0.00-52.2)	0.00 (0.00-35.6)	0.00	0.539
Savory snacks (g)	0.00 (0.00-29.1)	6.22 (0.00-41.7)	15.0	0.659
Sugar-sweetened beverage, SSB (ml)	267 (135-369)	332 (202-464)	336	0.474
Sweet snacks (g)	23.7 (3.83-67.8)	25.1 (0.00-51.3)	89.7	0.719
Vegetables (g)	115 (52.7-171)†	53.2 (32.2-95.4)†	117	0.012*
Whole grains (g)	0.00 (0.00-9.50)	0.00 (0.00-19.5)	0.00	0.787
**Nutrients**				
Total energy (kcal)	1860 (1710-2320)	1820 (1680-2240)	2410	0.439
Carbohydrates (% of total energy)	41.6 (37.9-49.8)†	49.4 (46.0-52.9)†	49.7	0.008*
Protein (% of total energy)	20.6 (16.7-22.6)†	16.3 (14.9-19.3)†	14.1	0.008*
Total fat (% of total energy)	38.2 (32.9-40.2)†	34.4 (31.0-37.2)†	33.2	0.053
Saturated fat (% of total energy)	12.4 (11.9-14.5)†	11.6 (9.96-13.4)†	13.4	0.111
Monounsaturated fat (% of total energy)	13.8 (11.6-15.0)	12.0 (10.3-14.2)	11.4	0.315
Polyunsaturated fat (% of total energy)	6.57 (5.91-7.86)	5.99 (5.32-7.99)	5.08	0.309
Beta-carotene (mcg per 1000 kcal)	0.18 (0.00-13.5)	0.56 (0.00-5.40)	12.3	0.603
Calcium (mg per 1000 kcal)	262 (215-311)	276 (198-319)	265	0.831
Cholesterol (mg per 1000 kcal)	207 (163-254)	191 (146-222)	91.3	0.115
Dietary fiber (g per 1000kcal)	6.26 (5.92-8.36)	6.95 (5.94-7.64)	7.06	0.449
Iron (mg per 1000 kcal)	5.88 (5.24-6.77)	6.73 (5.23-7.23)	5.46	0.526
Sodium (mg per 1000 kcal)	1760 (1320-2250)	1640 (1440-2040)	1570	0.648
Vitamin A (mcg per 1000 kcal)	289 (187-403)	250 (149-327)	227	0.499
**Eating behavior**				
Cognitive dietary restraint	14.0 (13.0-17.0)	16.0 (13.3-18.0)	17.0	0.626
Emotional eating	5.00 (3.00-8.00)	6.00 (4.25-7.75)	4.00	0.296
Uncontrolled eating	21.0 (18.0-24.5)	21.5 (19.0-23.8)	21.0	0.755

Data were presented as median (interquartile range: 25th-75th percentile). Differences between the 3 ethnic groups were analyzed using Kruskal-Wallis H test. Due to the small sample size for Indian subjects (n=3), no interquartile range is available and subsequent comparison was conducted between Chinese and Malay subjects using Mann-Whitney U test. Asterisk * denotes significant difference between the 3 ethnic groups with $p<0.05$, while † denotes significant difference between Chinese and Malay subjects with $p<0.05$.

Table S6. Food groups, nutrient intakes, and eating behavior between children/adolescents with MHO and children/adolescents with MUO (MS definition) stratified by race

	MS definition					
	Chinese			Malay		
	MHO (n=16)	MUO (n=5)	*p*	MHO (n=23)	MUO (n=5)	*p*
**Food groups**						
Deep fried food (g)	78.8 (40.8-126)	51.3 (44.4-217)	0.978	50.9 (19.1-126)	53.2 (37.9-128)	0.527
Fast food and processed convenience food (g)	33.3 (0.00-122)	101 (0.00-130)	0.825	84.0 (21.3-176)	46.7 (0.00-155)	0.410
Fish (g)	0.00 (0.00-4.65)	22.4 (9.17-121)	0.556	0.00 (0.00-48.7)	47.7 (0.00-63.5)	0.133
Fruits (g)	17.9 (0.00-56.1)	0.00 (0.00-120)	0.918	0.00 (0.00-37.4)	0.00 (0.00-20.9)	0.846
Savory snacks (g)	0.00 (0.00-27.8)	0.00 (0.00-65.5)	0.165	10.0 (0.00-33.3)	0.00 (0.00-102)	0.947
Sugar-sweetened beverage, SSB (ml)	279 (133-387)	240 (113-403)	0.410	313 (200-444)	433 (211-1130)	0.515
Sweet snacks (g)	23.5 (0.00-90.5)	24.9 (15.3-57.4)	0.790	25.2 (0.00-51.7)	25.0 (0.00-59.7)	0.708
Vegetables (g)	93.3 (51.6-159)	167 (82.2-275)	0.344	55.8 (33.3-95.7)	40.0 (6.33-89.7)	0.481
Whole grains (g)	0.00 (0.00-0.00)	0.00 (0.00-117)	1.000	0.00 (0.00-20.0)	9.67 (0.00-42.3)	0.792
**Nutrients**						
Total energy (kcal)	1850 (1670-2340)	1860 (1820-2770)	0.975	1900 (1670-2240)	1730 (580-2330)	0.694
Carbohydrates (% kcal)	41.9 (38.5-49.8)	39.9 (35.2-49.2)	0.889	49.8 (45.4-53.1)	49.0 (47.5-53.9)	0.918
Protein (% kcal)	18.1 (16.5-22.7)	21.9 (18.4-23.1)	0.869	16.5 (15.1-19.7)	15.3 (13.4-17.0)	0.201
Total fat (% kcal)	38.5 (32.8-39.8)	38.2 (32.4-41.7)	0.743	34.3 (31.0-37.6)	36.0 (29.9-38.0)	0.368
Saturated fat (% kcal)	12.4 (11.9-14.4)	12.7 (11.1-15.7)	0.621	11.6 (9.75-13.1)	13.2 (10.2-14.3)	0.478
Monounsaturated fat (% kcal)	13.7 (11.4-15.0)	14.0 (12.0-15.3)	0.957	12.0 (10.1-14.2)	13.2 (12.4-14.9)	0.423
Polyunsaturated fat (% kcal)	6.56 (5.91-8.13)	7.10 (5.58-7.31)	0.693	5.98 (5.22-8.30)	6.01 (5.06-7.18)	0.814
Beta-carotene (mcg per 1000 kcal)	0.09 (0.00-19.2)	0.81 (0.00-18.2)	0.981	2.37 (0.00-5.91)	0.00 (0.00-0.48)	0.640
Calcium (mg per 1000 kcal)	274 (225-343)	221 (176-259)	0.488	277 (196-350)	216 (200-292)	0.389
Cholesterol (mg per 1000 kcal)	207 (161-240)	207 (154-328)	0.993	186 (147-215)	196 (104-291)	0.515
Dietary fiber (g per 1000kcal)	6.46 (5.98-8.89)	6.02 (5.46-6.40)	0.393	7.06 (5.93-7.66)	6.67 (5.70-7.34)	0.543
Iron (mg per 1000 kcal)	5.83 (5.05-7.44)	5.97 (5.24-6.77)	0.770	6.91 (5.23-7.28)	6.04 (3.82-6.80)	0.356
Sodium (mg per 1000 kcal)	1850 (1320-2650)	1460 (1360-1860)	0.959	1580 (1430-2030)	1800 (1440-2050)	0.517
Vitamin A (mcg per 1000 kcal)	266 (182-404)	330 (141-374)	0.864	279 (145-328)	198 (157-287)	0.455
**Eating behavior**						

Cognitive dietary restraint	14.5 (13.3-16.8)	14.0 (12.5-17.0)	0.681	16.0 (14.0-18.0)	14.0 (12.5-17.5)	0.150
Emotional eating	6.00 (3.25-8.00)	5.00 (3.00-6.00)	0.610	6.00 (4.00-8.00)	6.00 (5.00-7.00)	1.000
Uncontrolled eating	22.0 (18.3-25.0)	20.0 (17.5-22.5)	0.452	22.0 (19.0-24.0)	21.0 (18.5-22.5)	0.574

Data were presented as median (interquartile range: 25th-75th percentile). Differences in continuous variables between groups were analyzed using quantile regression with adjustment for age, sex and BMI-SDS.
